# The role of stimuli of life events and sleep quality in the incidence of acute low-tone sensorineural hearing loss

**DOI:** 10.3389/fneur.2024.1480216

**Published:** 2024-11-15

**Authors:** Xi He, Chao Huang, Fan Jiang, Hongli Lan, Yu Huang, Maojie Liu, Dan Lai

**Affiliations:** ^1^Department of Otolaryngology Head and Neck Surgery, The Affiliated Hospital of Southwest Medical University, Luzhou, China; ^2^Department of Otolaryngology Head and Neck Surgery, Zigong First People’s Hospital, Zigong, Sichuan, China; ^3^Department of Otolaryngology Head and Neck Surgery, The Second Affiliated Hospital of Chengdu Medical College, Nuclear Industry 416 Hospital, Chengdu, Sichuan, China

**Keywords:** acute low-tone sensorineural hearing loss, life events, sleep quality, psychosomatic factor, retrospective study

## Abstract

**Introduction:**

It is difficult to detect acute low-tone sensorineural hearing loss (ALHL) because of only low-frequency hearing loss and atypical early symptoms. The etiology of ALHL is still elusive, and psychosomatic factors influence deafness and tinnitus. Therefore, this study aimed to clarify the correlation between psychosomatic factors and the incidence of ALHL to facilitate the prevention of ALHL.

**Methods:**

Patients with stuffy ears and tinnitus who were admitted to the Outpatient Clinic of the Affiliated Hospital of Southwestern Medical University (Luzhou, China) from July 2020 to May 2023 were identified in this retrospective study. The general data, hearing screening form, the Life Event Scale, and the Pittsburgh Sleep Quality Index scale were employed to assess patients’ hearing levels and stimuli of psychosomatic symptoms. Finally, the correlation among the stimuli of life events, sleep quality, and ALHL was statistically analyzed.

**Results:**

A total of 97 ALHL patients and 97 healthy participants were enrolled in the case group and control group, respectively. The two groups had no significant differences in general information (all *p* > 0.05). The amount of negative life event stimuli was significantly larger in ALHL patients than in the normal population (*p* = 0.000). Patients with ALHL had significantly poorer sleep quality than the healthy population (*p* = 0.000). There was a positive correlation between sleep quality and ALHL severity (250 Hz: *r* = 0.336, *p* = 0.001; 500 Hz: *r* = 0.299, *p* = 0.003), and a positive correlation between the stimuli of life events and sleep quality (*r* = 0.535, *p* = 0.000).

**Discussion:**

Sleep quality was found to be closely associated with the degree of hearing loss in ALHL patients, and there was also a strong correlation between sleep quality and the stimuli of life events. Therefore, psychosomatic factors may play an important role in the occurrence of ALHL.

## 1 Introduction

Acute low-tone sensorineural hearing loss (ALHL) was first identified and defined by Takashi Abe et al. in 1981 as a sudden hearing loss limited to frequencies below 1,000 Hz, with normal hearing in the remaining higher frequencies and without vestibular dysfunction (1). ALHL patients are clinically characterized by low-tone tinnitus, aural fullness, and hearing loss, which mainly involves women aged ≤40 years, and stuffy ears and tinnitus are the chief complaints, while hearing loss is difficult to be detected by patients themselves (2, 3). The exact pathogenesis of ALHL has not yet been fully clarified. A previous study demonstrated that it is a precursor of Meniere’s disease, because the two disease share several symptoms, such as tinnitus, sensorineural hearing loss, and occasional vertigo (2). Another study employed the glycerol test and electrocochleogram, and concluded that ALHL was caused by endolymphatic hydrops (3). However, ALHL showed good treatment outcomes and spontaneous recovery rates, and very few ALHL patients were eventually diagnosed with Meniere’s disease (4–6). Studies suggested that ALHL was originally a specific type of sudden deafness (7). However, the symptoms of ALHL are milder, accompanied by high spontaneous recovery rates, and the hearing loss at low frequencies does not meet the standard for sudden deafness. Thus, ALHL was treated as an independent disease for decades (2, 8).

Studies have mainly utilized the diagnostic criteria proposed by Toshiichi Imamura in 1997 for ALHL, which emphasized that the acoustic conduction resistance was associated with type A tympanogram without an obvious etiology or a clinically detectable etiology (8). The differences in diagnostic criteria were mainly attributable to the threshold requirements for hearing loss. In a recent study, basic consensus criteria were used with the frequencies below 1,000 Hz, in which frequencies of 250 and 500 Hz indicated hearing threshold ≥30 db, and three frequencies of 2,000, 4,000, and 8,000 Hz represented hearing threshold ≤20 db (9). However, the above diagnostic criteria all emphasize the idiopathic nature of ALHL, i.e., the absence of a definite or clinically detectable etiology.

Studies reported a wide range of ALHL-associated risk factors, including infections, cardiovascular insufficiency, autoimmune diseases, hyperviscosity syndrome, strokes, coronavirus disease 2019 (COVID-19), etc. (10–12). Recent studies have found that iron deficiency anemia and abnormal thyroid function are associated with the development of ALHL (13, 14). However, exposure to these factors does not necessarily indicate that patients may encounter aggravated symptoms. These findings and the inability to clinically detect the presence of organic ear lesions such as blood clotting, neurologic damage, and hair cell dysfunction in patients with ALHL have raised concerns about whether systemic factors are associated with the development of ALHL. A growing body of evidence supported that psychosomatic factors remarkably contributed to sudden hearing loss and tinnitus, such as stress, unemployment, depression, anxiety, etc., and the influences of these factors were reported to be particularly evident in the early stages of hearing loss and tinnitus (15–19). Scholars have reported a correlation between inadequate sleep quality and the onset of unilateral idiopathic sudden sensorineural hearing loss and tinnitus (20, 21). It was reported that life events might cause stress, which in turn may lead to illness and even create a vicious cycle with significant physical effects (22). Thus, it is reasonable to speculate that psychosomatic effects may play an important role in the onset of ALHL.

However, no study has explored the correlation between psychosomatic factors (e.g., stimuli of life events and sleep quality) and patients with ALHL before the onset of their symptoms. Whether the stimuli of life events or sleep disorders could trigger ALHL remains uncertain. Therefore, the present study aimed to investigate the association of psychosomatic factors with the development of ALHL by assessing the stimuli of life events and sleep quality in ALHL patients.

## 2 Materials and methods

### 2.1 Patients screening

Patients with ear disorders who were admitted to the Outpatient Clinic of the Affiliated Hospital of Southwestern Medical University (Luzhou, China) from July 2020 to May 2023 were enrolled in this retrospective study. The clinicians performed a routine audiological examination and completed an audiometric scale. The researchers then divided the subjects into case and control groups based on their hearing results. The inclusion criteria of case group were as follows: (1) Hearing-associated complaints and duration of illness lasting for about 2 weeks; (2) Intact tympanic membrane and type A acoustic conductance pattern in the affected ear, as well as the presence of stapedial muscle acoustic reflex; (3) Frequencies of 250 and 500 Hz indicating hearing threshold ≥30 db, in addition to frequencies of 1,000, 2,000, 4,000, and 8,000 Hz reflecting hearing threshold ≤20 db according to pure-tone testing; (4) Passing otoacoustic reflex test; (5) serious cognitive or intellectual impairment was excluded; (6) Exclusion of patients with hearing loss of clear etiology, such as Ménière’s disease, drugs, infection, and trauma. The control group consisted of middle-aged and young adult subjects from the health check-up center who required ENT examinations and had normal results from objective assessments, including otoscopy and pure-tone audiometry.

### 2.2 General data and hearing screening form

Basic information of the subjects, including gender, age, chief complaints, educational level, and marital status, was collected. All subjects underwent tympanometry, transient evoked otoacoustic emission (TEOAE), and pure-tone testing. Tympanometry was carried out using a middle ear analyzer (Grason-Stadler Inc., Eden Prairie, MN, USA). TEOAEs were performed using the AccuScreen (Madsen, Copenhagen, Denmark). The hearing thresholds of 250, 500, 1,000, 2,000, 4,000, and 8,000 Hz were tested by an audiogram (ASTERA; Otometrics, Copenhagen, Denmark). All of the above medical records were completed prior to patient grouping and the results of the hearing examinations were recorded on the hearing screening form.

The survey included the Life Events Scale (LES), and the Pittsburgh Sleep Quality Index (PSQI) scale. All the respondents were informed of the objectives of the questionnaire, and the collected data may be used in the future relevant studies. All subjects voluntarily accepted the questionnaire to review recently experienced adverse psychosomatic events after verbal informed consent. The subjects were not subjected to any intervention throughout the course of the study, and there were no follow-up or prospective issues.

### 2.3 The stimuli of life events (LES)

LES was developed by Dickson Yang and Yalin Zhang in 1986, which combined the widely used social readjustment scale in the United States and the actual situation in China. It has shown promising reliability and validity, in which a total of 48 items are classified into three dimensions: family life (28 items), work and study (13 items), and social and other events (7 items) (4). There were two additional items for respondents to add (life events that caused distress, while were not mentioned in the questionnaire). The questionnaire was designed to record life events within about 1 year (they were also recorded if a longer period was provided, whereas they were the subject’s main source of life stimulation). Life events were divided into positive life events (causing mania, etc.) and negative life events (causing anxiety, paranoia, depression, sadness, etc.). Besides, subjects could select the intensity of the stimulus according to their feelings (i.e., none, mild, moderate, severe, and very severe). Subsequently, the duration of the disturbance caused by the stimulus was selected, including 3 months, 6 months, 1 year, and long-term. Finally, the stimuli of life events were calculated using the following formula: number of events * degree of impact of the event * duration of the event; total number of stimuli = number of positive events + number of negative events.

### 2.4 Sleep quality (PSQI)

The Chinese version of the PSQI was employed, which had exhibited satisfactory pertinency and validity for the Chinese normal adults and patients with sleep disorders (23). It mainly assesses the subject’s sleep in the last month from 7 aspects: (1) Time to fall asleep: if the sleep time is longer than 30 min, the score can be accumulated; (2) Sleep duration: the time between sleep and getting up, which is inequal to the time in bed; (3) Sleep efficiency: = sleep time/time in bed * 100%; (4) Sleep questions: the score is calculated based on the situation in 9 sleep-associated questions; (5) Conscious sleep quality: based on the subject’s own feelings; (6) Use of hypnotic drugs: the higher the frequency, the higher the score; (7) Daytime dysfunction: evaluation of daytime life and work. Finally, the above-mentioned scores are calculated and the sleep grade is accordingly evaluated as follows: 0 ~ 5: satisfactory; 6 ~ 10: unsatisfactory; 11 ~ 15: poor; 16 ~ 21: very poor.

### 2.5 Statistical analysis

SPSS 26.0 software (IBM, Armonk, NY, USA) was utilized to perform the statistical analysis. The count data were expressed as percentile, normally distributed continuous variables by means and standard deviations, and others by medians and interquartile ranges. Normal distribution of continuous variables per group was assessed by Shapiro–wilk test. The Chi-square test was employed to compare the general information of subjects between the two groups and the Kolmogorov–Smirnov (K-S) Z test was employed to assess the association between life events simili and sleep quality between the two groups. In addition, Spearman’s rank correlation analysis was applied to explore the correlation among the degree of hearing loss, stimuli of life events, and sleep quality scores. A two-tailed *p* < 0.05 was considered statistically significant. Stepwise linear regression analysis was applied to test the correlation between assessed sleep quality and hearing loss, with a two-sided *p* less than 0.05 considered statistically significant.

## 3 Results

### 3.1 Clinical features of ALHL

A total of 97 patients who were diagnosed with ALHL were included in the case group, and 97 populations with normal hearing was enrolled in the control group. As presented in Table 1, the majority of patients with ALHL were female (about 72.2%). Half of them aged 40 years old or younger (66.0%). There was no significant difference in the prevalence of unilateral hearing loss between the left ear and the right ear (97.9% unilateral, 53.6% in the left ear). In addition, the vast majority of patients with ALHL (approximately 72.2%) had tinnitus as their chief complaint. The pure-tone testing revealed that most of the patients had mild and moderate levels of hearing loss (range, 30–60 db). The degree of hearing loss for ALHL patients was concentrated in the mild range (>25 db and ≤ 40 db), and very few patients had severe hearing loss (>60 db).

**Table 1:** Patients’ demographic and clinical data.

		Value	Ratio
Gender	Female	70	72.2%
	Male	27	27.8%
Age	0 ~ 40 years	64	66.0%
	>40 years	33	34.0%
Ear	Left	52	53.6%
	Right	43	44.3%
	Double	2	2.1%
Chief complaint	Tinnitus	70	72.2%
	Other	27	27.8%
Mean hearing loss in low-tone	30 ~ 40 dB	57	58.8%
	>40 dB	40	41.2%

### 3.2 General information of ALHL and normal groups

Table 2 describes the number, percentage and OR value of the subjects in the case and control groups. The mean age of the subjects in the two groups was 34.87 ± 12.77 and 35.49 ± 9.40, respectively, *χ*2 = 0.447, *p* = 0.656. Therefore, there were no significant differences between the two groups in gender, age, marital status and education.

**Table 2:** Comparison of case group and control group subjects.

	ALHL patients (*n* = 97)	Control group (*n* = 97)	OR (95%CI)	*p*
Gender				
Female	70 (72.2%)	63 (64.9%)	1.399 (0.761–2.573)	0.354
Male	27 (27.8%)	34 (35.1%)		
Degree of education				
Junior high school and below	27 (27.8%)	28 (28.9%)	0.951 (0.509–1.775)	1.000
High school and junior college	38 (39.2%)	43 (44.3%)		
Bachelor degree and above	32 (33.0%)	26 (26.8%)		
Married	63 (64.9%)	71 (73.2%)	0.679 (0.368–1.253)	0.277
Unmarried	34 (35.1%)	26 (26.8%)		
Negative events	81 (83.5%)	75 (77.3%)	1.485 (0.725–3.040)	0.366
Family events	62 (63.9%)	54 (55.7%)	1.411 (0.793–2.510)	0.305
Work and study events	70 (72.2%)	43 (44.3%)	3.256 (1.790–5.921)	0.000
Social and other events	27 (27.8%)	24 (24.7%)	1.173 (0.619–2.225)	0.744
PSQI				
0 ~ 5	12 (12.4%)	80 (82.5%)	33.333 (14.984–74.152)	0.000
6 ~ 10	55 (56.7%)	17 (17.5%)		
11 ~ 15	27 (27.8%)	0 (0%)		
16 ~ 21	3 (3.1%)	0 (0%)		

### 3.3 The stimuli of life events

**Figure 1:**
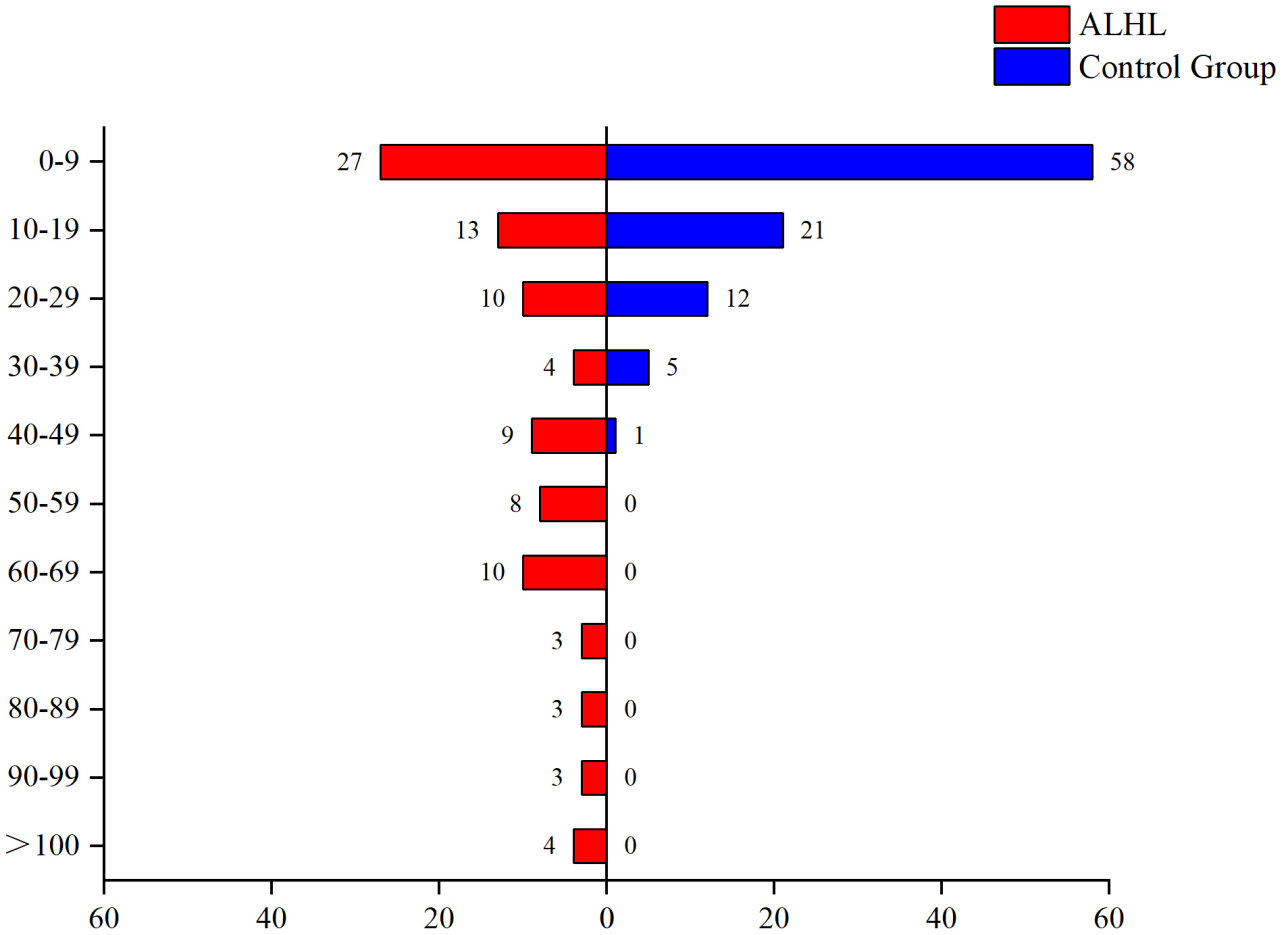
Between-group comparison of LES. The vertical coordinates represent LES scores, with the number of patients with ALHL in each score band on the left and the healthy people on the right.

The survey performed using the LES indicated that the vast majority of patients experienced psychiatric symptoms, such as anxiety, depression, paranoia, and mania. Only 16.5% of the patients reported that they did not experience malignant events that affected their quality of life. The results of the comparison between the case and control groups in the number and the percentage of subjects are demonstrated in Table 2. Although the number of subjects in the ALHL group was larger compared to the normal group, the stimuli were not statistically significant, except for the life events stimuli of work-study origin. Besides, the results of comparison between the two groups in the median negative life events stimuli are depicted in Figure 1. The vertical coordinates represent LES scores, with the number of patients with ALHL in each score band on the left and the healthy people on the right. The amount of negative life event stimuli was significantly larger in ALHL patients than that in the normal population (*Z* = 2.154, *p* = 0.000).

### 3.4 Sleep quality

**Figure 2:**
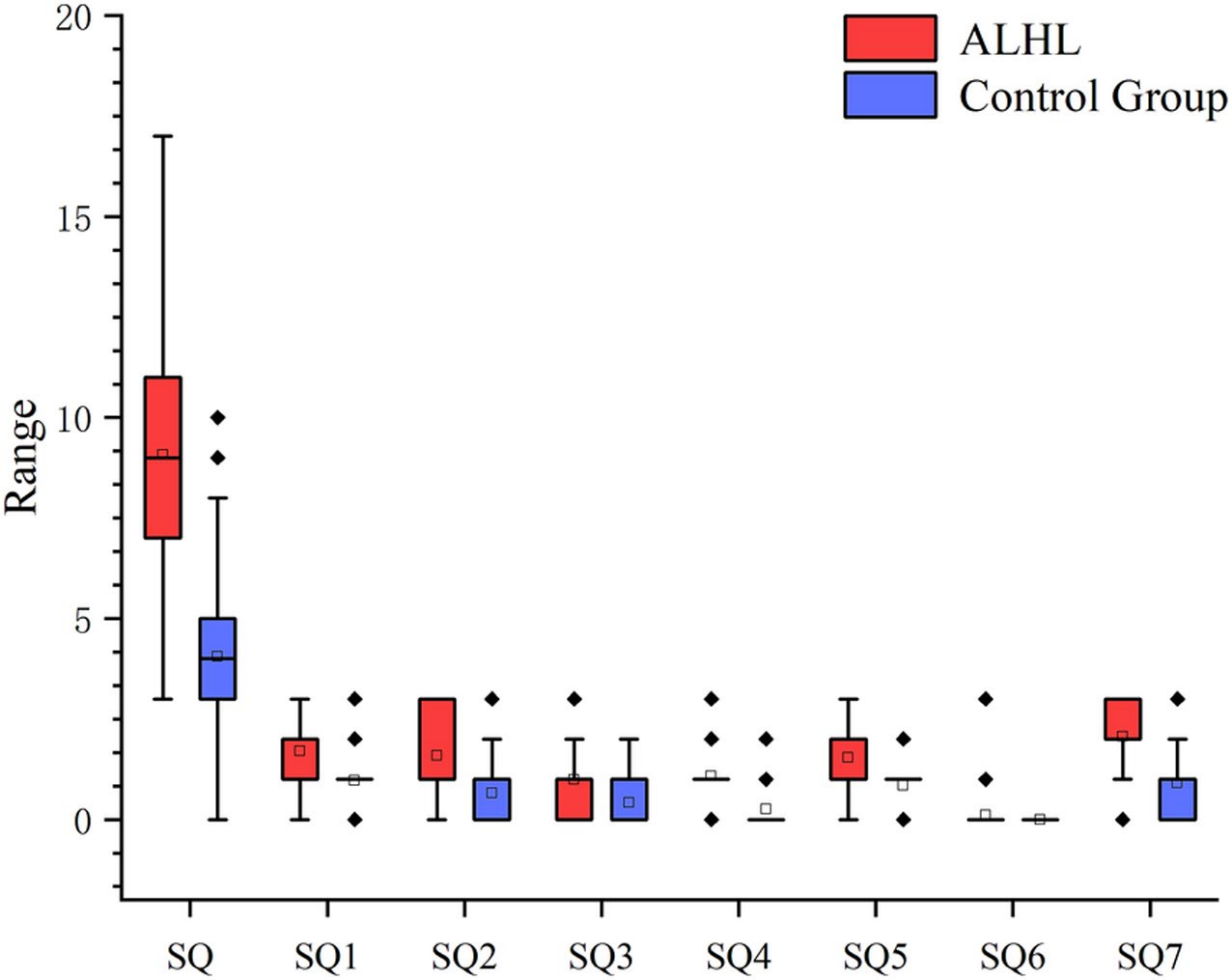
Between-group comparison of PSQI (median scores and IQR). It’s the results of the comparison between the two groups in terms of median PSQI scores in various categories and their IQR. SQ1, Time to fall asleep; SQ2, Sleep duration; SQ3, Sleep efficiency; SQ4, Sleep questions; SQ5, Conscious sleep quality; SQ6, Use of hypnotic drugs; SQ7, Daytime dysfunction.

According to the PSQI scale, it was found that 56.7% of the patients with ALHL had a moderate sleep quality within 1 month prior to searching for the medical assistance, and 27.8% of the patients had a poor sleep quality. The number, percentage and OR value of subjects in the case and control groups concerning the PSQI scores are shown in Table 2. The number of patients with ALHL who had good sleep quality was significantly smaller than that of the normal population, and there were ALHL patients with severe sleep quality disorders. Since the PSQI scores for each category do not follow a normal distribution, it is crucial to use a measure that minimizes the impact of extreme values on the results. By utilizing the median, we can achieve a more reliable representation of trends within the dataset, ensuring that findings are not skewed by outliers and accurately reflect the underlying patterns in sleep quality. As shown in Figure 2, the comparison of median PSQI scores between the two groups reveals that ALHL patients had worse sleep quality than the normal population, with significant differences in various categories (*Z* = 3.321, 2.513, 2.010, 3.590, 3.087, 3.877, 4.822; all *p* = 0.000), except for the use of hypnotic medication (*Z* = 0.646, *p* = 0.798). Furthermore, the results of the stepwise linear regression analysis are shown in Table 3. The results show that sleep quality has a significant effect on ALHL, and the values of adjusted *R*^2^ for 250 Hz and 500 Hz were 10.3 and 8%, respectively, with a statistically significant difference of *p* < 0.001 significance.

**Table 3:** Stepwise linear regression analyses with the PSQI as the dependent variable.

	*R* ^2^	Adjusted *R*^2^	Standardized beta coefficient	Beta significance
250 Hz hearing loss	0.113	0.103	0.336	<0.001
500 Hz hearing loss	0.090	0.080	0.3	<0.003

### 3.5 Correlation of ALHL with stimuli of life events and sleep quality

The correlation analysis of ALHL at 250 and 500 Hz with stimuli of life events and sleep quality was performed. The correlation coefficient was calculated to clarify the relationship (Table 4). Two datasets (*r* and *p*) are shown at the intersection of each item, where r is the correlation coefficient. It was revealed that there was a positive correlation between incidence of ALHL at 250 and 500 Hz and sleep quality index (250 Hz: *r* = 0.336, *p* = 0.001; 500 Hz: *r* = 0.299, *p* = 0.003). Thus, it could be concluded that the poorer sleep quality, the worse hearing loss at 250 and 500 Hz. No significant correlation was found between stimuli of negative life events and incidence of ALHL (250 Hz: *r* = 0.061, *p* = 0.551; 500 Hz: *r* = 0.011, *p* = 0.912). In addition, a positive correlation was found between stimuli of life events, especially negative life events, and sleep quality (total negative stimuli: *r* = 0.535, *p* = 0.000), demonstrating that as the stimuli of negative life events increased and worsened, sleep quality became worse.

**Table 4:** The correlation of the incidence of ALHL with life events and sleep quality.

		Total negative events	Sleep quality	Hearing loss at 250 Hz	Hearing loss at 500 Hz
Total negative events	*r*		0.535^**^	0.061	0.011
	*p*		0.000	0.551	0.912
Sleep quality	*r*	0.535^**^		0.336^**^	0.299^**^
	*p*	0.000		0.001	0.003
250 Hz hearing loss	*R*	0.061	0.336^**^		0.671^**^
	*p*	0.551	0.001		0.000
500 Hz hearing loss	*R*	0.011	0.299^**^	0.671^**^	
	*p*	0.912	0.003	0.000	

r, Spearman’s rho; **Correlation is significant at the 0.01 level (two-tailed).

## 4 Discussion

A previous study showed that ALHL mainly affects women aged less than 40 years unilaterally, and most mainly complain of tinnitus or stuffy ears (2). The results of the present study are consistent with the above-mentioned manifestations, thus, the epidemiological characteristics of patients with ALHL may be not different significantly across ethnic groups and geographic regions, and the current diagnostic criteria for ALHL may have satisfactory generalizability and accuracy worldwide. In addition, hearing loss in ALHL patients was concentrated in the mild-to-moderate range, only in the low-frequency range of less than 1,000 Hz, which was mainly distributed in non-verbal frequency ranges, indicating that hearing loss is less likely to be detected compared to low-pitched tinnitus or stuffy ears. Additionally, due to the relatively mild hearing loss observed in patients with ALHL, tests such as otoacoustic emissions (with a positive result defined as >30 dB) typically show no significant abnormalities. Furthermore, the widely recognized high self-recovery rate in these patients substantiates the classification of ALHL as distinct from other hearing loss conditions associated with cochlear damage.

It was found that almost all ALHL patients had experienced psychological or psychiatric events, mainly negative life events. ALHL patients have significantly higher LES scores than the healthy population, indicating that there might be some correlations between them. However, comparisons of exposure rates to life event stimuli, as well as correlation analyses showed invalidity of the finding that the greater the stimuli of the life events, the more severe the degree of hearing loss in ALHL patients. Besides, life events mostly originated from the work and study (and is likely to be associated with the incidence of ALHL), followed closely by family events. Combined with the above-derived characteristics of the onset of ALHL, i.e., predominantly present in young and middle-aged women (aged less than 40 years old). The reason may be that ALHL patients need to constantly switch between the roles of family and work to meet the demands of both roles, especially married women and women who already have children, imposing more mental stress on them (24). In addition, the design of the scale in the three sections (family life, work and study, social and other events) may contribute to this effect (the two sections have the greatest weight coefficients).

It is found that ALHL patients usually suffer from sleep disorders. The onset of ALHL tends to be developed within 2 weeks, while the PSQI investigated their sleep status at 1 month prior to seeking medical assistance, thus, a decrease in sleep quality is a predisposing factor for ALHL. Comparisons of both sleep disorder exposure and sleep quality scores were significantly higher in patients with ALHL than in the healthy population, and correlation analyses all showed the sleep quality was closely correlated with the severity of hearing loss in ALHL patients, in which the worse the sleep quality, the more severe the hearing loss. However, the reason for the apparent discrepancy in the use of hypnotic drugs may be due to the fact that people are squeamish about the adverse drug reactions, so they do not readily try hypnotic drugs even if they have sleep disorders. According to previous studies, a poor sleep quality can cause mental stress, which in turn may lead to the development of diseases, such as chronic tinnitus (25, 26), which is recognized as one of the main symptoms of ALHL. Specifically, mental stress increases the release of steroids through excitation of the hypothalamus and excites the sympathetic nervous system, which may together lead to the increased excitement in the body (25, 27). Eventually, prolonged stress in the body may affects the function of auditory cells (28), leading to ALHL, and we, in the present study, hypothesized that the prolongation of this endocrine and neurological synergy could lead to stress, the more severe the dysfunction of auditory cells, and therefore, the more severe the hearing loss in ALHL patients. In addition, the poor sleep quality may cause anxiety and hyperarousal (29). During the daytime, the lack of sleep leads to a decrease in the efficiency of work and life, and the overwrought state continues during the daytime, which may further affect the sleep quality at night. As a result, individuals with sleep disorders are in daytime and nighttime hyperarousal states, and the body cannot eventually bear such a heavy burden, which may be an important factor in triggering ALHL. Moreover, the poorer the sleep quality, the more overwrought the person becomes, and the more pronounced the patient’s experience of tinnitus and stuffy ears.

Additionally, the present study revealed that there was a positive correlation between life events and sleep quality scores. The more stimuli caused by life events, the worse the sleep quality, and vice versa. It may be attributed to the fact that negative life events and negative coping styles can lead to the lower sleep quality, and sleep disturbances at night may result in physical and mental disorders that interfere with normal daytime life, which in turn leads to more negative coping styles toward life events (30, 31). Thus, these two factors interact with each other, eventually leading to the development of the disease like ALHL. Therefore, the close association between the two makes it possible to simultaneously assess them, enabling us to comprehensively and accurately assess the patient’s overall mental psychological status. The effects of negative life events may still be within the psychological tolerance of ALHL patients, but when such effects cause sleep disturbances, the combined influence of the two will be magnified, which ultimately leads to a positive correlation between sleep quality and ALHL severity. The present study found the positive correlation is weak which may because that current study is only a preliminary exploration, the association is tangible, but a targeted questionnaire on the influence of psychiatric factors on hearing needs to be further development.

Through this study, we have reason to believe that it is necessary to pay attention to the psychosomatic state, emotional problems and sleep disorders of patients with ALHL in addition to conventional drug treatment in the clinic, and carry out targeted intervention to reduce the psychiatric stress of patients and improve the quality of sleep, which will lead to better therapeutic effects. The primary limitation of this study lies in its retrospective design, which may have introduced bias due to the inability to control for exposures and outcomes. Furthermore, confounding factors, such as comorbidities or lifestyle influences, as well as regional diversity, were not considered, potentially affecting the validity of the results. Given that the study was conducted in a single country and at a single center, future research should not only address the retrospective nature and potential confounding variables but also incorporate more diverse sampling across regions to mitigate biases stemming from economic and socio-cultural disparities.

## 5 Conclusion

Significant exposure rate differences and severity correlations were found between hearing loss and poor sleep quality in patients with ALHL, indicating that the poor sleep quality was a risk factor for the development of ALHL and was closely associated with the degree of hearing loss in ALHL. But the stimuli of life events may not significantly contribute to the pathogenesis of ALHL.

## Data Availability

The datasets presented in this study can be found in online repositories. The names of the repository/repositories and accession number(s) can be found in the article/supplementary material.

## References

[1] Abe T Kon Y Murai K Tsuiki T. Clinical pictures of low tone sudden deafness. Nihon Jibiinkoka Gakkai kaiho. (1988) 91:667–76. PMID: 3418437

[2] Park MJ Kim SH Kim SS Yeo SG. Clinical characteristics and short-term outcomes of acute low frequency sensorineural hearing loss with Vertigo. Clin Exper Otorhinol. (2018) 11:96–101. doi: 10.21053/ceo.2017.00948, PMID: 29310430 PMC5951067

[3] Yamasoba T Sugasawa M Kikuchi S Yagi M Harada T. An electrocochleographic study of acute low-tone sensorineural hearing loss. European archives of Oto-rhino-laryngology: Official journal of the European Federation of Oto-Rhino-Laryngological Societies (EUFOS): Affiliated with the German Society for Oto-Rhino-Laryngology-Head and Neck Surgery. Germany: European archives of oto-rhino-laryngology and head & neck (1993) 250:418–422. 8286108 10.1007/BF00180389

[4] Lin Y Wang JL Sun F Shen JJ Wang ZX Qiu JH. Recurrent low frequency sensorineural deafness. J Clin Otorhinol Head Neck Surg. (2018) 32:474–6. doi: 10.13201/j.issn.1001-1781.2018.06.019 29737749

[5] Fushiki H Junicho M Kanazawa Y Aso S Watanabe Y. Prognosis of sudden low-tone loss other than acute low-tone sensorineural hearing loss. Acta Otolaryngol. (2010) 130:559–64. doi: 10.3109/00016480903311245, PMID: 19916896

[6] Kim H Lee H Kim Y-C Park E Choi J Rah YC. Update on clinical manifestation in acute low-tone hearing loss. Korean J Otorhinol Head Neck Surg. (2020) 63:403–8. doi: 10.3342/kjorl-hns.2019.00759

[7] Editorial Board of Chinese Journal of Otorhinolaryngology Head and Neck Surgery; Society of Otorhinolaryngology Head and Neck Surgery, Chinese Medical Association. Guideline of diagnosis and treatment of sudden deafness (2015). Zhonghua Er Bi Yan Hou Tou Jing Wai Ke Za Zhi. (2015) 50:443–7. 26695792

[8] Imamura S Nozawa I Imamura M Murakami Y. Clinical observations on acute low-tone sensorineural hearing loss. Survey and analysis of 137 patients. Ann Otol Rhinol Laryngol. (1997) 106:746–50. doi: 10.1177/000348949710600906, PMID: 9302905

[9] Im GJ Kim SK Choi J Song JJ Chae SW Jung HH. Analysis of audio-vestibular assessment in acute low-tone hearing loss. Acta Otolaryngol. (2016) 136:649–54. doi: 10.3109/00016489.2016.1152506, PMID: 26963446

[10] Young YH. Contemporary review of the causes and differential diagnosis of sudden sensorineural hearing loss. Int J Audiol. (2020) 59:243–53. doi: 10.1080/14992027.2019.1689432, PMID: 31714154

[11] Meng X Wang J Sun J Zhu K. COVID-19 and sudden sensorineural hearing loss: a systematic review. Front Neurol. (2022) 13:883749. doi: 10.3389/fneur.2022.883749, PMID: 35572936 PMC9096262

[12] Chau JK Lin JR Atashband S Irvine RA Westerberg BD. Systematic review of the evidence for the etiology of adult sudden sensorineural hearing loss. Laryngoscope. (2010) 120:1011–21. doi: 10.1002/lary.20873 20422698

[13] Taki M Hasegawa T Ninoyu Y Mohri H Hirano S. Low-frequency sensorineural hearing loss associated with Iron-deficiency Anemia. J Int Adv Otol. (2021) 17:465–7. doi: 10.5152/iao.2021.9369, PMID: 34617900 PMC8975401

[14] Chen L Wang YJ Sun X Zhang N Li YN Fan ZM. Analysis of prognostic factors of low-frequency type of sudden sensorineural hearing loss. Zhonghua er bi yan hou tou jing wai ke za zhi. (2020) 55:652–7. doi: 10.3760/cma.j.cn115330-20191212-00756, PMID: 32668873

[15] Schmitt C Patak M Kröner-Herwig B. Stress and the onset of sudden hearing loss and tinnitus. Int Tinnitus J. (2000) 6:41–9. PMID: 14689617

[16] Shan A Ting JS Price C Goman AM Willink A Reed NS. Hearing loss and employment: a systematic review of the association between hearing loss and employment among adults. J Laryngol Otol. (2020) 134:387–97. doi: 10.1017/S0022215120001012, PMID: 32468973

[17] Fowler EP Fowler EP Jr. Somatopsychic and psychosomatic factors in tinnitus, deafness and vertigo. Ann Otol Rhinol Laryngol. (1955) 64:29–37. doi: 10.1177/000348945506400106 14362314

[18] Konzag TA Rübler D Bandemer-Greulich U Frommer J Fikentscher E. Psychological comorbidity in subacute and chronic tinnitus outpatients. Z Psychosom Med Psychother. (2005) 51:247–60. doi: 10.13109/zptm.2005.51.3.247, PMID: 16276478

[19] Schüssler G Geishauser E Rüger U. Psychosomatic factors in idiopathic sudden deafness. HNO. (1992) 40:4–9. PMID: 1568883

[20] Lu T Li S Ma Y Lai D Zhong J Li G. Positive correlation between tinnitus severity and poor sleep quality prior to tinnitus onset: a retrospective study. Psychiatry Q. (2020) 91:379–88. doi: 10.1007/s11126-019-09708-2, PMID: 31925625

[21] Wang YJ Wang MM Hou ZQ Fang ZM Wang HB. Sleep quality analysis in patients with unilateral idiopathic sudden sensorineural hearing loss. Lin chuang er bi yan hou tou jing wai ke za zhi. (2018) 32:209–13. doi: 10.13201/j.issn.1001-1781.2018.03.013 29775024

[22] Rabkin JG Struening EL. Live events, stress, and illness. Science (New York, NY). (1976) 194:1013–20. doi: 10.1126/science.790570 790570

[23] Kumar A Handa R Upadhyaya SK Gupta SJ. Validation of Hindi version of the Pittsburg sleep quality index. J Assoc Physicians India. (2021) 69:11–2. PMID: 34470185

[24] Tang CS. The influence of family-work role experience and mastery on psychological health of Chinese employed mothers. J Health Psychol. (2009) 14:1207–17. doi: 10.1177/1359105309342302, PMID: 19858340

[25] Mazurek B Szczepek AJ Hebert S. Stress and tinnitus. HNO. (2015) 63:258–65. doi: 10.1007/s00106-014-2973-7 25862619

[26] Betz LT Mühlberger A Langguth B Schecklmann M. Stress reactivity in chronic tinnitus. Sci Rep. (2017) 7:41521. doi: 10.1038/srep41521, PMID: 28134346 PMC5278380

[27] Mazurek B Boecking B Brueggemann P. Association between stress and tinnitus-new aspects. Otol Neurotol. (2019) 40:e467–73. doi: 10.1097/MAO.0000000000002180, PMID: 30870382

[28] Fujinami Y Mutai H Mizutari K Nakagawa S Matsunaga T. A novel animal model of hearing loss caused by acute endoplasmic reticulum stress in the cochlea. J Pharmacol Sci. (2012) 118:363–72. doi: 10.1254/jphs.11227FP, PMID: 22362185

[29] Bonnet MH Arand DL. Hyperarousal and insomnia: state of the science. Sleep Med Rev. (2010) 14:9–15. doi: 10.1016/j.smrv.2009.05.002 19640748

[30] Zarenoe R Hällgren M Andersson G Ledin T. Working memory, sleep, and hearing problems in patients with tinnitus and hearing loss fitted with hearing aids. J Am Acad Audiol. (2017) 28:141–51. doi: 10.3766/jaaa.16023 28240981

[31] Ren Z Zhang X Shen Y Li X He M Shi H. Associations of negative life events and coping styles with sleep quality among Chinese adolescents: a cross-sectional study. Environ Health Prev Med. (2021) 26:85. doi: 10.1186/s12199-021-01007-2, PMID: 34481463 PMC8418725

